# Diurnal changes of retinal microvascular circulation and RNFL thickness measured by optical coherence tomography angiography in patients with obstructive sleep apnea–hypopnea

**DOI:** 10.3389/fendo.2022.947586

**Published:** 2022-08-09

**Authors:** Yi Cai, Wen-Bo Liu, Miao Zhou, Yu-Tong Jin, Guo-Sheng Sun, Long Zhao, Fang Han, Jin-Feng Qu, Xuan Shi, Ming-Wei Zhao

**Affiliations:** ^1^ Department of Ophthalmology, Peking University People’s Hospital; Eye Diseases and Optometry Institute, Beijing Key Laboratory of Diagnosis and Therapy of Retinal and Choroid Diseases, College of Optometry, Peking University Health Science Center, Beijing, China; ^2^ Department of Biostatistics and Bioinformatics, Emory University, Atlanta, GA, United States; ^3^ Sleep Medicine Center, Department of Respiratory and Critical Care Medicine, Peking University People’s Hospital, Beijing, China

**Keywords:** optical coherence tomography angiography, obstructive sleep apnea-hypopnea, retinal nerve fiber layer, deep capillary plexus, radial peripapillary capillary, vessel density, ocular diurnal changes

## Abstract

**Purpose:**

To evaluate capillaries perfusion and retinal nerve fiber layer (RNFL) thickness diurnal changes of macular/optic disc regions among participants with or without obstructive sleep apnea-hypopnea (OSA) using spectral-domain optical coherence tomography angiography (OCTA).

**Methods:**

In this study, we enrolled a cohort of 35 participants including 14 patients with mild-to-moderate OSA, 12 patients with severe OSA, and 9 healthy individuals. All participants had Berlin questionnaire filled. At 20:00 and 6:30, right before and after the polysomnography examination, a comprehensive ocular examination was conducted. The systemic and ocular clinical characteristics were collected, and OCTA scans were performed repeatedly. Blood flow and RNFL thickness parameters were then exported using built-in software and analyzed accordingly.

**Results:**

After sleep, the overall vessel density (VD) variables, especially macular and choriocapillaris VDs, were relatively comparative and stable. One exception was the RPC vessel density at the inside-disc region with a decreasing trend in the mild-to-moderate group (p=0.023). RNFL changes before and after sleep in the nasal-inferior and peripapillary region were statistically significant (p=0.003; p=0.043) among three groups. And multiple testing correction verified the significant difference in diurnal changes between the mild-to-moderate group and the control group in pairwise comparisons (p=0.006; p=0.02).

**Conclusions:**

The changes of imperceptible blood flow and RNFL thickness overnight around optic disc areas could be observed in OSA patients. Despite physiological fluctuations, aberrant diurnal changes might be useful for identifying a decrease in micro-environmental stability associated with the development of various ocular diseases such as glaucoma. Other VD variables, especially macular and choriocapillaris VDs, are relatively stable in eyes of patients having OSA with different severity.

## Introduction

Obstructive sleep apnea–hypopnea (OSA) is a highly prevalent systemic disorder characterized by episodes of obstruction or collapse of the upper airway and subsequent partial (hypopnea) or complete (apnea) cessation of airflow for more than 10 s despite the remaining respiratory effort. It is frequently related to aberrant blood-gas changes and is always associated with anomalous sleep patterns and cortical arousal (1). It is the most common type of sleep disorder in adults, and the prevalence is 3.62% for adults over 30 years in Shanghai, China, with a still increasing prevalence consistent with the rising trend of obesity (2, 3). Abnormal activation of the sympathetic nervous system and elevated serum catecholamines are the initiating factors for many vascular, metabolic, and inflammatory diseases (4, 5); furthermore, OSA is also the main cause of life quality impairment and is associated with an increased risk of traffic accidents. The high incidence and severe impact on life quality make it a huge burden on public health.

OSA has already been verified to be highly associated with neurological disorders such as stroke, depression, headache, elevated cranial pressure, and peripheral neuropathy (6). The higher incidence of neurological disorders in patients with OSA is thought to be possibly related to the frequency of apnea-induced hypoxemia (4). Besides, OSA can lead to various optic nerve impairments (7, 8), including non-arteritic ischemic optic neuropathy (NAION) and glaucoma, as well as pathological involvement of the retinal/choroidal tissue, such as retinal vein occlusion (RVO) (9, 10) and pachychoroidal diseases like central serous chorioretinopathy (CSC) and polypoidal choroidal vasculopathy (PCV) (9, 11). In the last few years, a large number of scientific reviews, meta-analyses, and articles have been published to verify the significant association between OSA and glaucoma/CSC in adults (12–15), serving as the evidence of a strong association with the development of certain eye diseases.

Up to now, the pathogenesis between OSA and ocular optic nerve-related diseases or pachychoroid diseases is still unclear (9), probably associated with the abnormal hypoxia-induced factors upregulation, hypercoagulable state, oxidative stress, and endothelial cell damage secondary to hypoxia episodes (9). The related studies are mainly of clinical nature because of the long period for animal modeling. Thanks to the transparency of retinal tissue, the microvasculature of the retina tissue is the only microvascular network that can be directly observed. With the advancement of OCTA technology in the last decade, we can perform not only morphological but also quantitative studies on retinal microvasculature (16) and speculate on the pathogenic mechanism of OSA *in vivo*.

Quantitative assessment of microvascular circulation changes in patients with OSA is scarce. The first study of quantitative OCTA measurements in patients with OSA was performed by Yu et al. in 2017, who concluded that vascular density (VD) in the macular and retinal regions differed significantly among groups classified by OSA severity. The severe group with an apnea–hypopnea index (AHI) of more than 30 breaths/hour had significantly lower VD in the peripapillary area. In addition, vessel densities in the parafoveal and peripapillary regions were significantly negatively correlated with AHI (17). However, Laura Moyal et al. (18) reported that OCTA parameters at the SCP level were comparable between groups regardless of the severity of OSA, although significant functional variations in FDT-Matrix™ and Humphrey 24/2 were noted, suggesting that changes in functional examinations might occur before blood flow fluctuation emerged. Contradictory results emerged from these existing studies. Therefore, in our previous study, we also took VD in the deep capillary plexus (DCP) into account and performed another cross-sectional comparison of quantitative OCTA parameters in patients with OSA of different severities. We found that the severe group had significantly higher VD in parafoveal and perifoveal regions at the level of DCP, whereas the mild-to-moderate group had a significantly lower radial peripapillary capillary (RPC) VD in the peripapillary area. We considered that blood flow changes in the macula may first appear in the DCP layer in patients with OSA in the cross-sectional comparison. Reduced VD in the peripapillary region of the RPC layer may lead to subsequent RNFL changes (19). However, considering that complex compensatory mechanisms might exist and lead to adaptive modulation of retinal microvasculature and tissues during chronic OSA, immediate circadian retinal microvascular changes after exposure to hypoxia attacks and sympathetic system activation may be more responsive to the instant ocular changes over a short period. However, studies on altered circadian OCT or OCTA parameters are relatively lacking.

Therefore, in this study, we investigated the diurnal variation of nerve fiber layer thickness near the optic disc and the microvascular VD in the optic disc and macular regions in OSA patients.

## Methods

This prospective observational study consisted of 26 consecutive participants newly diagnosed with OSA and nine age-matched controls, all recruited from the Sleep Laboratory of the People’s Hospital of Peking University. The study was performed between January 2018 and September 2020 and was approved by the ethics committee at mentioned university. The protocols adhered to the aims of the Declaration of Helsinki. All subjects participated voluntarily, and their written informed consent was obtained.

The general experimental method has been elaborated in the previous study (19). Participants were enrolled according to the following criteria: 1) aged between 25 and 65 years; 2) this was the first time of sleep monitoring and had no history of any sleep disorder; 3) no history of any ocular surgery or trauma; 4) no history or family history of primary glaucoma; and 5) spherical equivalence <-6.0 D. The exclusion criteria were as follows: 1) already received any treatment for OSA including continuous positive airway pressure (CPAP); 2) with history of coexisting systemic diseases, including diabetes, migraine, hypertension, coronary heart diseases, or already recently treated by any vasoactive medication; 4) with an axial length out of the range of 22.0 to 26.5 mm; 5) combined with other ocular diseases or previous intraocular surgery history, including amblyopia, macular degeneration, CSC, optic nerve diseases, retinal detachment, and uveitis; 6) compromised OCTA signal secondary to refractive media opacity; 7) poor cooperation in OCTA examinations due to inability to control eye movements like strabismus; 8) best-corrected visual acuity (BCVA, converted to LogMAR scores) greater than 0.1.

After filling out the Berlin questionnaire, ocular data were collected at around 20:00. Baseline demographic data including, sex, age, current cigarette smoking status, neck circumference, body mass index (BMI), and blood pressure were also documented. The examiner and the participants were unaware of the polysomnography (PSG) results at the time of receiving the ocular and body examination. The next morning after PSG, the same set of OCTA scans was repeated as soon as the PSG test was completed (at around 6:30 a.m.). The morning blood pressure and intraocular pressure (IOP) were also collected.

### Ocular examination

All participants received comprehensive ocular examinations, including BCVA (transformed into LogMAR score), IOP measured using a non-contact tonometer (TX-F fully automated IOP meter, Canon Canada), anterior segment examination, fundus examination, axial length and central corneal thickness (CCT) measured using the optical method (ZEISS IOLMaster 500, Carl Zeiss Meditec, Jena, Germany), and OCTA scans (Optovue RTVue XR Avanti, Optovue Inc., Fremont, CA). All images and data were evaluated by the same expert examiner (YC). To ensure consistency of the longitudinal assessment, topical mydriatic eye drop was not applied and all OCT scans were performed inside a silent room with a few minutes of dark adaptation.

### Optical coherence tomography angiography

We preferred the right eye as the study eye unless there were obvious motion artifacts difficult to eliminate despite repeating. High-definition (HD) imaging was conducted using the RTVue XR Avanti Spectral Domain OCT System (version 2017,1,0,155), which had been described in detail in our previous study (19). The macula area was imaged in a 6 mm × 6 mm square, and the corresponding flow data were directly processed based on the split-spectrum amplitude-decorrelation angiography (SSADA) algorithm. Vascular density was defined as the proportion of blood vessels in the corresponding segmental scan. The foveal region was surrounded by the parafoveal area, which was defined as a ring area with an inner radius of 0.5 mm and an outer radius of 1.5 mm (20, 21). The perifoveal region was another annulus area with a width of 1.5 mm outside the parafoveal region (19, 22). The superficial capillary plexus (SCP) was generated as the layer between 3 µm below the internal limiting membrane (ILM) to 15 µm below the inner plexiform layer (IPL), while the DCP was defined as the area from 15 to 70 µm below the IPL (19, 23, 24). The innermost borderline of the avascular zone (FAZ) at the level of SCP was automatically identified through the built-in software. The area of the FAZ, VD within the FAZ zone, and VD of the area 300 μm beyond FAZ were also obtained from the integrated software AngioAnalytics. The optic nerve head VD of the RPC layer was analyzed separately after the acquisition of 4.5 mm × 4.5 mm scans. The inside-disc zone was defined as an elliptical region corresponding to the boundary of the optic nerve. The peripapillary region was defined as the 2–4-mm annulus region extending from the inside-disc region and automatically divided into eight zones (Temporal Superior, Superior Temporal, Superior Nasal, Nasal Superior, Nasal Inferior, Inferior Nasal, Inferior Temporal, and Temporal Inferior, abbreviated as GH-TS, GH-ST, GH-SN, GH-NS, GH-NI, GH-IN, GH-IT, and GH-TI sections, respectively) according to the modified Garway-Heath (GH) grids (25). RPCs, the most superficial capillary layer, refer to the capillaries sited from the inner limiting membrane with an offset of 0 μm to the base of the nerve fiber layer, which corresponds to the thickness of the entire nerve fiber layer. The macular choriocapillaris vessel density was obtained using the built-in flow measurement tool by calculating the mean ratio of the vascular area occupied in a circle of 36 mm^2^ centered on the foveola (26). Each OCTA scan mentioned above was performed two times, and for quantitative parameters, the mean value of the corresponding area was calculated for subsequent analysis.

### Polysomnography

All subjects were monitored using a standard polysomnography system (Alice 6 LDx; Philips Respironics, Andover, MA) with standard nocturnal polysomnography continuous electroencephalography recorded from 10 p.m. to 6 a.m. (Alice 6 LDx; Philips Respironics, Andover, MA). All subjects underwent nocturnal polysomnography (PSG) for at least 6 h in a quiet, individual room. The definition of apnea events obeys the sleep guidelines. The AHI (times/h) was assessed for each patient based on Academy of Sleep Medicine standards and used as the indicator of the severity of OSA (27). Participants diagnosed with OSA (AHI >5 times/h) (27) were divided into two groups according to the severity of OSA: participants with an AHI of 5 to 30 times/h were classified in the mild-to-moderate group; subjects with an AHI over 30 times/h were graded into the severe group. Subjects verified by an AHI of 5 or less served as the control group.

### Statistical analysis

Statistical analyses were performed using R (version 4.0.3). Continuous data were expressed as mean ± SD (min, max). The Shapiro–Wilk test was used to verify normality for continuous data. One-way analysis of variance (ANOVA) was used to perform a cross-sectional comparison between three groups for normal distribution data; otherwise, Kruskal–Wallis was used as an alternative, and Tukey *post-hoc* tests were used for pairwise comparison. Circadian valuable change was analyzed using paired t-test or paired-sample Wilcoxon signed-rank test. For two continuous measurements of the same scan item, the intraclass correlation coefficient (ICC) was calculated to determine the consistency of the corresponding continuous variables. Correlation coefficients between sectional RNFL diurnal difference and other parameters were analyzed and adjusted in the multiple regression analysis. p-values <0.05 were considered statistically significant.

## Results

A total of 14 subjects diagnosed with mild-to-moderate OSA, 12 subjects with severe OSA, and nine control subjects without a diagnosis of OSA were included in the final analysis. The systemic and ocular parameters regarding age, blood pressure, BMI, AL, CCT, and intraocular pressure that may affect the analysis of quantitative OCTA data were all comparable. The characteristics of the subjects’ systemic and ocular parameters are shown in **Table 1**.

**Table 1:** Systemic and ocular characteristics of subjects.

Variables	Control group (N = 9)	Mild-to-moderate group (N = 14)	Severe group (N = 12)	P value
Age (y)	41.78 ± 12.13 (25, 60)	37.29 ± 8.00 (31, 61)	43.33 ± 9.06 (28, 56)	0.260
Sex				
Female	5 (55.6%)	2 (14.3%)	2 (16.7%)	
Male	4 (44.4%)	12 (85.7%)	10 (83.3%)	
Eye				
OD	8 (88.9%)	13 (92.9%)	12 (100%)	
OS	1 (11.1%)	1 (7.1%)	0 (0%)	
Cigarette smoking (%)	2 (22.2%)	3 (21.4%)	3 (25%)	
BMI (kg/m^2^)	23.64 ± 2.88 (18.60, 28.34)	25.39 ± 3.01 (20.55, 31.64)	28.56 ± 4.68 (21.88, 39.45)	0.058^†^
AHI (events/h)	2.76 ± 1.02 (1.00, 4.00)	17.20 ± 5.43 (9.70, 27.20)	54.98 ± 15.57 (34.90, 87.40)	0.000* ^†^
Lowest value of SpO_2_ (%)	89.67 ± 6.52 (74.00, 95.00)	85.50 ± 4.55 (75.00, 93.00)	66.75 ± 10.94 (50.00, 84.00)	0.000*^†^
Nocturnal systolic pressure (mmHg)	116.33 ± 11.75 (100.00, 135.00)	118.43 ± 12.28 (102.00, 140.00)	118.33 ± 15.13 (85.00, 140.00)	0.923
Nocturnal diastolic pressure (mmHg)	74.33 ± 9.70 (60.00, 85.00)	77.21 ± 9.29 (64.00, 90.00)	77.58 ± 10.14 (50.00, 90.00)	0.717^†^
Morning systolic pressure (mmHg)	112.56 ± 12.20 (90.00, 128.00)	116.57 ± 10.94 (100.00, 140.00)	120.17 ± 15.19 (88.00, 142.00)	0.923
Morning diastolic pressure (mmHg)	74.67 ± 7.94 (60.00, 84.00)	77.64 ± 8.13 (64.00, 94.00)	78.75 ± 11.53 (54.00, 92.00)	0.415
Nocturnal MAP (mmHg)	88.33 ± 9.45 (73.33, 101.67)	90.95 ± 9.69 (78.67, 106.00)	91.17 ± 11.18 (61.67, 104.67)	0.788
Morning MAP (mmHg)	87.30 ± 8.55 (70.00, 98.67)	90.62 ± 8.69 (78.67, 108.00)	92.56 ± 12.35 (65.33, 108.67)	0.501
Nocturnal MOPP (mmHg)	46.56 ± 6.89 (37.33, 56.78)	46.42 ± 6.66 (38.44, 60.67)	48.19 ± 9.97 (21.11, 59.89)	0.476^†^
Morning MOPP (mmHg)	46.31 ± 7.03 (30.67, 52.78)	46.44 ± 6.26 (37.33, 55.89)	49.70 ± 9.01 (28.56, 62.56)	0.471
Diurnal difference in MOPP (mmHg)	-0.25 ± 3.67 (-6.67, 4.44)	0.02 ± 4.10 (-6.11, 7.84)	1.51 ± 5.26 (-5.67, 11.00)	0.601
Neck circumference (cm)	34.67 ± 3.35 (31.00, 42.00)	38.36 ± 3.08 (32.00, 43.00)	39.17 ± 3.64 (34.00, 46.00)	0.012*
AL (mm)	23.82 ± 1.04 (22.28, 25.27)	24.66 ± 1.17 (22.47, 26.00)	24.66 ± 0.79 (23.04, 25.84)	0.118
CCT (µm)	545.00 ± 48.35 (432.00, 609.00)	537.36 ± 32.39 (488.00, 598.00)	541.58 ± 35.71 (454.00, 594.00)	0.892
Nocturnal IOP (mmHg)	12.33 ± 2.35 (9.00, 16.00)	14.21 ± 2.55 (10.00, 19.00)	12.58 ± 3.85 (8.00, 20.00)	0.259
Morning IOP (mmHg)	11.89 ± 2.85 (8.00, 16.00)	13.97 ± 2.21 (11.00, 17.00)	12.00 ± 3.10 (8.00, 17.00)	0.109^†^

Values are expressed as mean ± SD (range) or no. (%) according to the variable types; † represents using Kruskal–Wallis rank test for analysis; * and bold values represents for data with p < 0.05;

BMI, body mass index; AHI, apnea–hypopnea index; CCT, central corneal thickness; AL, axial length; Lowest value of SpO_2_, the lowest value of oxyhemoglobin saturation during sleep; IOP, intraocular pressure; MAP, mean arterial pressure, calculated by 2/3×diastolic blood pressure + 1/3×systolic blood pressure; MOPP, mean ocular perfusion pressure, calculated by 2/3×MAP – IOP.

Considering the prevalence of nerve fiber layer defect in OSA patients in the previous studies as mentioned above, we first investigated the retinal nerve fiber layer thickness changes in different peripapillary subdivisions corresponding to the Garway-Heath mapping of the VF in the range of 2–4-mm dual-ring area centered on the optic disc among different groups. The results suggested that the mean total peripapillary RNFL thickness in the baseline examination before PSG showed a decreasing trend in the OSA group compared to the control group, although without any statistical significance. In contrast, in certain Garway-Heath subdivisions, there were significant differences in the mean RNFL thickness at baseline, including the inferior hemisection (p = 0.048) and IT section (p = 0.009), respectively. In the longitudinal observation of circadian nerve fiber layer thickness changes using paired t-test analysis, the severe group was found to have significant differences between nocturnal and morning RNFL thickness in both the peripapillary, GH-S-Hemi, and GH-IN sections (p = 0.003; p = 0.003; p = 0.049, respectively), with the thickness being significantly higher in the morning than measured at night. In contrast, there were no significant diurnal differences of other sections in other groups (**Table 2**).

**Table 2:** RNFL parameters results of participates.

Valuables	Control group (N = 9)	Mild-to-moderate group (N = 14)	Severe group (N = 12)	Control group (N = 9)	Mild-to-moderate group (N = 14)	Severe group (N = 12)	p	p1	p2	p3
	Baseline (night)			Morning						
Peripapillary RNFLT (µm)	124.55 ± 8.99 (114.79, 139.00)	118.03 ± 13.75 (95.54, 141.79)	115.79 ± 6.36 (108.46, 124.80)	127.42 ± 7.90 (117.01, 138.68)	117.65 ± 13.92 (97.58, 143.28)	117.69 ± 7.83 (108.68, 134.78)	0.090^†^	0.760	0.572	0.003*^†^
GH-S-Hemi RNFLT (µm)	123.43 ± 11.19 (112.70, 144.47)	118.40 ± 13.88 (96.48, 141.58)	117.49 ± 7.27 (105.65, 131.56)	126.34 ± 8.95 (114.23, 142.88)	118.16 ± 14.43 (98.92, 144.38)	119.72 ± 8.45 (107.65, 136.23)	0.458	0.941	0.736	0.003*^†^
GH-I-Hemi RNFLT (µm)	125.76 ± 7.73 (115.86, 135.24)	117.61 ± 15.13 (94.52, 142.02)	113.96 ± 7.13 (104.14, 128.37)	128.61 ± 7.74 (117.42, 139.39)	117.10 ± 15.57 (96.15, 143.17)	115.49 ± 8.49 (105.40, 133.23)	0.048*^†^	0.995	0.506	0.092^†^
GH-NS RNFLT (µm)	115.60 ± 11.91 (103.51, 140.41)	113.63 ± 27.84 (80.17, 180.94)	109.83 ± 12.88 (90.41, 134.98)	118.34 ± 8.43 (103.86, 135.53)	112.26 ± 29.49 (77.90, 183.33)	110.99 ± 13.58 (87.48, 134.67)	0.863^†^	0.084	0.440	0.724^†^
GH-NI RNFLT (µm)	89.07 ± 9.64 (68.66, 102.02)	94.15 ± 22.40 (63.57, 133.32)	84.20 ± 10.90 (62.27, 97.97)	94.39 ± 6.40 (83.21, 102.72)	91.15 ± 25.56 (52.15, 128.44)	86.37 ± 12.05 (64.11, 105.38)	0.493^†^	0.613	0.529†	0.072
GH-IN RNFLT (µm)	159.37 ± 20.59 (134.29, 198.85)	149.71 ± 25.82 (107.93, 196.71)	148.49 ± 10.67 (132.89, 173.85)	163.03 ± 21.45 (132.50, 202.11)	150.38 ± 27.82 (110.74, 205.65)	151.90 ± 10.45 (138.54, 179.86)	0.523^†^	0.796	0.588	0.049*
GH-IT RNFLT (µm)	172.06 ± 15.07 (150.75, 196.65)	150.61 ± 18.57 (105.95, 178.17)	146.91 ± 18.70 (120.47, 182.43)	174.22 ± 17.29 (144.26, 198.90)	152.58 ± 17.47 (110.48, 177.21)	147.58 ± 18.82 (124.75, 187.42)	0.009*^†^	0.385	0.107	0.555
GH-TI RNFLT (µm)	87.02 ± 17.80 (69.86, 119.83)	75.51 ± 11.74 (54.67, 92.26)	77.14 ± 11.72 (62.49, 105.46)	86.31 ± 17.75 (71.20, 116.79)	75.05 ± 11.38 (58.94, 90.09)	76.23 ± 10.65 (63.57, 94.37)	0.320^†^	0.895	0.600	0.733^†^

Values are expressed as mean ± SD (range); † represents using the Kruskal–Wallis rank test for independent analysis, or Wilcoxon signed-rank test for paired analysis; p: p value of baseline (night) variables among three groups; p1: p value for diurnal comparison of paired variables in the control group; p2: p value for diurnal comparison of paired variables in the mild-to-moderate group; p3: p value for diurnal comparison of paired variables in the severe group; * and bold values represents for data with p < 0.05.

RNFLT, retinal nerve fiber layer thickness; GH, Garway-Heath-based grids. Temporal Superior, Superior Temporal, Superior Nasal, Nasal Superior, Nasal Inferior, Inferior Nasal, Inferior Temporal, and Temporal Inferior, abbreviated as GH-TS, GH-ST, GH-SN, GH-NS, GH-NI, GH-IN, GH-IT, GH-TI sections, respectively.

RNFL thickness referred to the mean RNFL thickness in the ellipsoidal area outward the boundary of the nipple area referring to optic nerve based on the modified Garway-Heath of a 2–4-mm grid. The RNFL thickness was corrected for ocular magnification.

The vascular densities at the level of SCP, DCP, and choriocapillaris in the macular area, RPC in different areas around the optic disc, FAZ area, and superficial FAZ VD were all comparable between the different groups. Vessel density in the areas and the corresponding layers mentioned above also maintained relatively diurnal stability, except for RPC vessel density in the inside-disc area: a significant decrease in RPC layer vessel density at the inside-disc area in the mild-to-moderate group occurred after an overnight sleep with apnea events emerging (p = 0.023). ICC of RPC VD at the inside-disc area revealed 0.80 (95% confidence interval: 0.644–0.896). Comparative and statistical analysis results are demonstrated in **Table 3**.

**Table 3:** OCTA and FAZ parameters of subjects.

Variables	Control group(N = 9)	Mild-to-moderate group (N = 14)	Severe group (N = 12)	Control group(N = 9)	Mild-to-moderate group (N = 14)	Severe group (N = 12)	p	p1	p2	p3
	Baseline (night)			Morning						
**Macular**										
SCP										
S Whole Image VD (%)	51.07 ± 2.67 (47.41, 54.15)	48.96 ± 2.49 (44.20, 52.20)	50.70 ± 3.23 (46.36, 54.95)	48.44 ± 3.98 (43.03, 53.87)	49.04 ± 3.29 (42.22, 54.00)	49.21 ± 4.69 (36.80, 54.86)	0.155	0.079	0.942	0.284
S Fovea VD (%)	17.38 ± 6.64 (6.05, 27.87)	18.48 ± 6.39 (9.93, 31.07)	18.58 ± 7.64 (5.59, 28.16)	16.36 ± 7.16 (8.33, 31.16)	17.99 ± 6.30 (10.33, 32.90)	18.17 ± 8.31 (6.45, 29.70)	0.912	0.337	0.495	0.585
S ParaFovea VD (%)	54.25 ± 3.38 (47.80, 58.09)	52.77 ± 3.46 (45.08, 57.22)	54.60 ± 3.38 (46.45, 58.64)	51.66 ± 3.94 (44.79, 57.67)	52.58 ± 2.81 (47.93, 56.05)	53.38 ± 4.71 (40.83, 59.28)	0.247^†^	0.114	0.884	0.419
S PeriFovea VD (%)	51.69 ± 2.86 (47.48, 55.13)	49.46 ± 2.72 (44.41, 52.97)	51.30 ± 3.68 (46.30, 56.20)	49.10 ± 4.01 (43.19, 54.54)	49.57 ± 3.67 (40.61, 54.81)	49.88 ± 5.11 (36.35, 56.01)	0.259^†^	0.101	0.926	0.335
DCP										
D Whole Image VD (%)	50.66 ± 5.87 (42.95, 57.67)	48.77 ± 4.25 (39.97, 55.68)	51.57 ± 4.97 (43.62, 58.69)	45.69 ± 8.49 (34.48, 58.92)	49.43 ± 5.20 (41.87, 61.72)	49.71 ± 6.28 (39.83, 58.67)	0.351	0.146	0.652	0.384
D Fovea VD (%)	32.64 ± 9.69 (15.12, 45.93)	35.12 ± 7.11 (24.74, 50.84)	37.11 ± 7.59 (25.39, 49.36)	32.24 ± 11.77 (14.98, 53.61)	35.04 ± 6.40 (27.96, 50.81)	36.13 ± 7.68 (25.48, 47.82)	0.454	0.793	0.902	0.110
D ParaFovea VD (%)	56.29 ± 4.13 (49.67, 61.56)	55.18 ± 2.89 (47.97, 59.47)	57.08 ± 3.23 (51.80, 61.24)	52.57 ± 5.55 (45.87, 60.24)	55.29 ± 2.82 (51.12, 61.12)	54.92 ± 4.03 (48.60, 60.53)	0.360	0.116	0.909	0.095
D PeriFovea VD (%)	51.84 ± 6.69 (42.66, 59.54)	49.94 ± 4.85 (40.46, 57.40)	53.21 ± 5.80 (42.92, 61.39)	46.73 ± 9.11 (33.89, 60.58)	50.41 ± 5.74 (41.82, 63.17)	51.37 ± 7.08 (40.74, 61.81)	0.351	0.160	0.769	0.435
Overall QI	7.89 ± 1.05 (6.00, 9.00)	7.50 ± 0.52 (7.00, 8.00)	7.75 ± 0.62 (7.00, 9.00)	7.22 ± 0.83 (6.00, 8.00)	7.71 ± 0.91 (6.00, 9.00)	7.75 ± 0.97 (6.00, 9.00)	0.393^†^	0.746^†^	0.336^†^	1.000
**FAZ**										
FAZ Area (mm^2^)	0.33 ± 0.16 (0.12, 0.59)	0.31 ± 0.10 (0.11, 0.44)	0.30 ± 0.10 (0.16, 0.46)	0.33 ± 0.16 (0.09, 0.60)	0.31 ± 0.10 (0.11, 0.44)	0.30 ± 0.10 (0.16, 0.48)	0.841	0.765	0.114	0.699
Perimeter night	2.18 ± 0.55 (1.43, 3.01)	2.10 ± 0.36 (1.26, 2.62)	2.09 ± 0.37 (1.47, 2.61)	2.16 ± 0.57 (1.25, 3.04)	2.13 ± 0.38 (1.31, 2.62)	2.09 ± 0.38 (1.51, 2.70)	0.877	0.454	0.164	0.982
Acircularity Index	1.10 ± 0.04 (1.06, 1.18)	1.09 ± 0.02 (1.06, 1.14)	1.08 ± 0.02 (1.05, 1.11)	1.10 ± 0.03 (1.07, 1.17)	1.09 ± 0.02 (1.06, 1.13)	1.08 ± 0.02 (1.05, 1.13)	0.542^†^	0.447	0.273	0.878
FD-300 Area VD (%)	54.04 ± 5.59 (45.72, 63.01)	53.95 ± 4.00 (46.67, 58.74)	55.99 ± 2.95 (50.67, 60.48)	52.13 ± 5.83 (42.37, 61.16)	54.34 ± 3.89 (46.04, 60.63)	53.98 ± 3.87 (48.98, 60.84)	0.409	0.051	0.705	0.130
FD-300 LVD (%)	12.84 ± 1.45 (10.45, 14.59)	12.60 ± 1.14 (9.83, 13.76)	13.29 ± 1.05 (11.62, 14.79)	12.11 ± 1.35 (9.85, 14.24)	12.56 ± 1.71 (10.63, 17.53)	12.73 ± 1.52 (9.39, 14.97)	0.355	0.089	0.943	0.622†
**Optic disc**										
Whole Image RPC VD (%)	52.15 ± 2.38 (48.91, 56.51)	50.86 ± 2.48 (47.26, 53.72)	50.74 ± 1.94 (47.34, 53.68)	52.18 ± 1.53 (50.46, 54.92)	49.98 ± 2.05 (46.44, 53.48)	50.62 ± 2.49 (45.56, 54.68)	0.320	0.970	0.065	0.806
Inside Disc RPC VD (%)	53.75 ± 3.82 (48.21, 61.93)	54.78 ± 5.05 (43.69, 63.39)	54.27 ± 3.92 (46.84, 59.97)	52.89 ± 4.75 (47.93, 64.20)	52.91 ± 5.02 (41.77, 61.65)	54.10 ± 3.88 (46.61, 61.69)	0.860	0.371	0.023*	0.885
Peripapillary RPC VD (%)	55.30 ± 2.16 (52.27, 59.81)	53.24 ± 2.41 (48.47, 56.61)	54.33 ± 2.71 (49.06, 58.26)	55.19 ± 2.63 (52.47, 59.36)	52.68 ± 2.51 (48.68, 56.45)	53.58 ± 3.15 (48.06, 58.27)	0.158	0.894	0.152	0.288
QI	8.22 ± 1.30 (5.00, 9.00)	8.14 ± 0.86 (7.00, 9.00)	8.25 ± 0.62 (7.00, 9.00)	8.22 ± 0.97 (7.00, 9.00)	8.00 ± 0.78 (6.00, 9.00)	8.33 ± 0.49 (8.00, 9.00)	0.721^†^	0.746^†^	0.530^†^	0.766^†^
**Choriocapillaris**										
Choriocapillaris VD (%)	70.40 ± 1.75 (66.67, 72.11)	70.87 ± 2.07 (67.00, 73.94)	70.41 ± 2.51 (66.00, 75.25)	69.58 ± 2.22 (65.72, 72.33)	70.71 ± 2.16 (67.33, 74.42)	70.44 ± 1.89 (67.67, 73.81)	0.826	0.117	0.728	0.948

Values are expressed in form of mean ± SD (range); † represents using Kruskal–Wallis rank test for independent analysis or Wilcoxon signed-rank test for paired analysis; p: p value of baseline (night) variables among three groups; p1: p value for diurnal comparison of paired variables in the control group; p2: p value for diurnal comparison of paired variables in the mild-to-moderate group; p3: p value for diurnal comparison of paired variables in the severe group; * and bold values represents for data with p < 0.05.

SCP, superficial capillary plexus; DCP, superficial capillary plexus; S, superficial; D, deep; RPC, radial peripapillary capillaries; VD, vessel density; LVD, length density; QI, quality index; FD-300, vessel density in the 300-µm region beyond the FAZ boundary.

**Figure 1:**
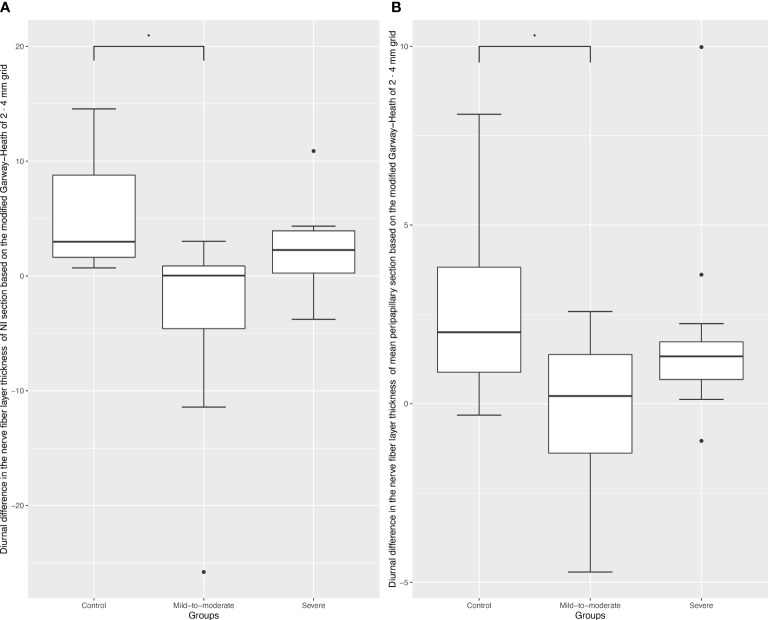
Box plots of diurnal RPC vessel density difference in different optic disc areas. Black dots indicate outliers (lower margin 5%, upper margin 5%). **(A)**, Box plots of diurnal RPC vessel density difference in the Garway-Heath-based-nasal inferior section; * refers to a Tukey *post-hoc* adjusted p value of 0.006. **(B)**, Box plots of diurnal RPC vessel density difference in the Garway-Heath-based-peripapillary section; * refers to a Tukey *post-hoc* adjusted p value of 0.02.

Comparisons of diurnal difference in OCTA vessel density and RNFL parameters between the three groups revealed that RNFL changes in the GH-NI and peripapillary regions were significantly different (p = 0.003; p = 0.043, respectively) among the three groups, and *post-hoc* tests verified a significant difference in diurnal changes between the mild-to-moderate group and the control group (p = 0.006; p = 0.02, respectively), which is demonstrated in **Figure 1**. ICC showed high reliability respectively (ICC = 0.94, 95% confidence interval: 0.89–0.971; ICC = 0.85, 95% confidence interval: 0.715–0.919).

Correlation analysis of the diurnal difference changes in RNFL thickness in the above two regions showed that nocturnal IOP and AL were significantly correlated with RNFL changes in the GH-NI region (p = 0.050; 0.021, respectively), but none of them was significant after being adjusted in the multiple linear regression (demonstrated in **Table 4**).

**Table 4:** Spearman’s rank correlation analysis/multiple linear regression analysis of systemic and ocular factors associated with diurnal difference in sectional RNFLT.

Spearman’s correlation analysis for diurnal difference in peripapillary RNFLT			Spearman’s correlation analysis for diurnal difference in GH-NI RNFLT			Multiple linear regression analysis for diurnal difference in GH-NI RNFLT		
Variables	β	p	Variables	β	p		β′	p′
AHI	-0.182	0.295	AHI	-0.192	0.268			
Age	-0.005	0.978	Age	-0.010	0.956			
BMI	0.098	0.575	BMI	0.076	0.665			
Nocturnal systolic pressure	-0.0845	0.628	Nocturnal systolic pressure	-0.101	0.564			
Nocturnal diastolic pressure	-0.194	0.263	Nocturnal diastolic pressure	-0.116	0.507			
Morning systolic pressure	-0.062	0.724	Morning systolic pressure	0.078	0.658			
Morning diastolic pressure	-0.159	0.362	Morning diastolic pressure	0.002	0.991			
Neck circumference	-0.054	0.760	Neck circumference	-0.036	0.836			
AL	-0.263	0.127	AL	-0.331	0.050*		-0.532	0.234
Nocturnal IOP	-0.354	0.057	Nocturnal IOP	-0.415	0.021*		-0.399	0.129
Morning IOP	-0.040	0.821	Morning IOP	-0.233	0.177			

* and bold values represents data with a p value < 0.05. β: Spearman’s rank correlation coefficient; β′: correlation coefficient in multiple linear regression analysis; p′: p value for the corresponding correlation coefficient in multiple linear regression analysis.

VD, vessel density; RNFLT, retinal nerve fiber layer thickness; GH, Garway-Heath-based grids; AL, axial length; IOP, intraocular pressure; AHI, apnea–hypopnea index; BMI, body mass index; NI, nasal inferior, I-Hemi, Inferior hemi; RPC, radial peripapillary capillaries; CCT, central corneal thickness.

## Discussion

In our previous study, we performed a cross-sectional analysis in subjects with OSA of different severity and age-matched controls and found spatial heterogeneity of VD changes in OSA patients (19). To further observe the immediate effects after apnea events, in the present study we investigated the diurnal variation of relevant RNFL/OCTA parameters in subjects with OSA of different severity and found significant diurnal VD changes at the RPC layer in the inside-disc region in the mild-to-moderate group rather than other groups, as well as intergroup differences in diurnal changes of the RNFL thickness at certain sections.

In the cross-sectional comparison of RNFL thickness around the optic disc area, we found a decreasing trend in mean peripapillary RNFL thickness among three groups, although this decreasing trend was not statistically significant, which could probably be attributed to the relatively small sample size. A statistically significant decrease in RNFL thickness occurred only in the GH-inferior and GH-IT sections, similar to previous studies. In our previous research, average peripapillary RNFL thickness was significantly different between different OSA groups, although no significant difference was observed in the *post-hoc* analysis (19). Sagiv et al. measured RNFL thickness changes in 62 moderate and 46 severe OSA participants in comparison to 108 age-matched controls using OCT. The results revealed that the mean, upper, lower, and temporal quadrants of RNFL thickness were significantly lower in the OSA group (28).

However, longitudinal observational studies on RNFL thickness in patients with OSA are relatively scarce. The decreasing trend of RNFL could also be reflected during the long-term follow-up. Mehmet Ozgur Zengin et al. found that the mean RNFL thickness at the last measurement (Month 12) was significantly lower in OSA patients than that of the first measurement and in controls during the 1-year follow-up of OSA patients and proposed that AHI was significantly correlated with retinal nerve fiber layer thickness (29). Besides, there was only one previous study on the diurnal variation of RNFL thickness in OSA patients: Chirapapaisn et al. proposed that RNFL thickness in the morning was significantly higher than in the evening in moderate OSA in the diurnal comparison (30). They speculated that swelling of RNFL might occur secondary to hypoxia episodes, increasing the cerebral spinal fluid pressure, and chronic hypercapnia and subclinical papilledema could only be detected on OCT images. Similar findings were also obtained in the present study, where we found a significant increase in the peripapillary RNFL thickness measured in the morning in the severe OSA group rather than in other groups, although similar changes were not seen in the mild-to-moderate group. We consider that the analogous result in the severe group may also be attributed to decreased optic nerve perfusion secondary to repetitive mechanical obstruction of the upper airway and recurrent hypoxic episodes in severe OSA. This process might initiate retinal nerve fiber ischemia and subtle swelling, leading to slightly but significantly increased nerve fiber layer thickness in the early morning. In a three-group comparison of the difference in diurnal variation, we found a significant decrease in the diurnal difference at the peripapillary and GH-NI regions in the mild-to-moderate group compared to the control group. We consider that the different changes present in the mild-to-moderate group versus the control group may be related to regulation of blood flow in mild OSA subjects. In our previous study, it was confirmed that the mild-to-moderate group had a significantly lower VD in the peripapillary region on the RPC network (19). Yu et al. also demonstrated the decrease in VD of the peripapillary area in the moderate group when compared to the normal-to-mild group. Nerve fiber layer thickness remained relatively stable during the daytime, but there were some small diurnal rhythmic changes, although hardly statistically significant, probably related to superficial retinal blood flow changes. Lisanne J. Balk et al. revealed that the significant physiological slight variation in the RNFL thickness may be relevant to altered hemodynamic status after exercise and dehydration (31). Farideh Sharifipour et al. also documented non-significant slight changes in various parameters including RNFL thickness in the control group, with the highest values at 9 a.m. and lowest at 3p.m., although none of them showed a significantly absolute change over the daytime (32). These mentioned studies demonstrated that there might be very small and insignificant physiological diurnal fluctuations in RNFL thickness which could only be observed shortly after awakening in the physiological state, and in patients with mild-to-moderate OSA, such physiological fluctuations are significantly compromised probably because of abnormal nocturnal activation of the sympathetic system. The diameter of the retinal arterioles has also been verified to be narrowest in the nasal inferior arcade, which may be the reason for more significant impact in the GH-NI region rather than in other sections (33). In contrast, the significant increase of sectional RNFL thickness in awakening in patients with severe OSA may be the result of mild edema in the nerve fiber layer.

No research on the VD diurnal change in OSA was ever analyzed. In the present study, we did not find any significant diurnal variable in macular regions at the level of either SCP or DCP. VDs in macular regions were all comparable in three groups, regardless of the time collected. This was consistent with the previous research regarding the macular regions in normal participants (34, 35). We speculated that this might be due to the unique autoregulation mechanism of retinal microvascular circulation. The contradiction between the heavy demand and limited ocular total blood flow is addressed through the delicately structured bilayer macular capillary network and auto-regulation strategy (36), to nurture multiple layers of the cells effectively while maintaining the gradient tension of oxygen in different layers of retina tissue, which has been already mentioned in our previous work (19). During the hypoxia and hypercapnia episodes, vasoconstriction of arteries followed by the excretion of ET-1, a known powerful vasoconstrictor, reduces the choroidal large blood flow subsequently. The choroid tissue is richly innervated by parasympathetic and sympathetic nerve fibers (37), while retinal vessels branched from the central retinal artery are not. Autoregulation of blood flow is achieved mainly through perceiving changes in local oxygen/carbon dioxide concentration, which is also called metabolic coupling (36, 37). Besides, to compensate for this change in blood flow, vasomotion through small arterioles in SCP (myogenic mechanism) would increase the effective utilization of limited blood flow, and a bilayer microvessel network would also provide a buffer when coping with fluctuations in blood flow (38). In our previous study, we had found that compared to the control group, the severe group had significantly higher VD in the parafoveal and perifoveal regions at the level of deep capillary plexus (DCP), indicating that blood flow changes in macular areas might first appear at the DCP layer in OSA patients (19), probably due to long-term adaptive compensation. However, the metabolic coupling regulation mechanism is sensitive; we speculated that macular VD changes within 12 h between night and morning could be negligible, especially in the patient without pathological vascular changes such as atherosclerosis or hypertension.

Regarding VD in the optic disc areas, we found that the RPC vessel density at the inside-disc region decreased significantly in the mild-to-moderate group in the morning, although the decline was slight, while other groups remained relatively stable during the day and night. In our previous cross-sectional study, we had noticed that VD decreased significantly in the moderate-mild group compared to the control group in the peripapillary region on the radial peripapillary capillary (RPC) network (19). The same conclusion was also noted in various previous studies (18, 39, 40). Yu et al. also mentioned that, compared to the parafoveal area, VD reduction was more pronounced in the peripapillary area (17). Although the baseline peripapillary VD measurements at night were relatively comparable among the three groups, which may be due to the relatively smaller population in the present study, after at least a 6-h-long sleep and overnight PSG examination, inside-disc VD demonstrated a significant difference in the mild-to-moderate group. However, the diurnal VD difference calculated in disc areas was also comparable in the three groups. We speculated that, compared to macular regions, VD changes in disc areas were more vulnerable to the total blood flow changes, especially in the RPC layer, which nurtures nerve fiber bundles sited in the RNFL layer of the inner retina. This might be attributed to the radial distribution of microvascular in the RPC layer. They radiated directly out of the central retinal artery in the optic nerve head section; in this way, VD at the level of the RPC layer in disc areas will be directly influenced by the blood flow changes. However, the residual time for retaining the change in optic disc blood flow did not last as long as RNFL thickness changes did.

In the analysis of blood flow changes at the level of choriocapillaris in the macular area, no significance was noticed between the three groups, and VD differences between day and night were also comparable. As mentioned above, the choroid tissue is richly innervated by autonomic vasoactive innervation and thus vulnerable to blood flow change in larger vessels and activation of the sympathetic nervous system, however, it can also regulate blood flow quickly at the same time. In one meta-study analysis of CSCR and OSA, findings suggested that patients with CSCR were more likely to have OSA, while patients with moderate/severe OSA had decreased subfoveal choroidal measurements on EDI-OCT scans (41). Besides, He et al. also reported that blood supply in choroid significantly decreased in moderate and severe OSA patients (42), Serpil Yazgan et al. (43) also noted that the peripapillary choroidal thickness of all quadrants was significantly thinner in the moderate and severe subgroups of OSA, and central macular choroidal thickness was significantly thinner in all subgroups of OSA subjects (43). However, Mehmet Özgür Zenginet al. (44) demonstrated that no significant variations in choroidal thickness were observed between patients with OSA and healthy subjects. Emine Esra Karaca et al. (45) also found no significant correlation between the severity of OSA and choroidal thickness in macular areas in a prospective study of 74 OSA patients and 33 normal subjects. They concluded that patients with OSA were able to protect choroidal thickness despite hypoxia to some extent. In our study, we also did not observe any change in the diurnal choriocapillaris VD analysis. We speculated that changes in choriocapillaris blood flow in patients with different severities of OSA did not have a relatively clear trend like VD and RNFL changes in disc areas. We considered that it could be related to fast vascular self-regulatory capacity in the early onset of OSA especially in young patients.

This study has several limitations. First, the subjects included in the study were relatively young and the population was small. Given the large number of examinations performed on every subject, to control the total time of the ophthalmic examination for better cooperation, and to meet the independence condition in statistical analysis, we performed OCTA measurements in only one eye, which further affects the sample size, although there was no evidence of differences in choosing different eyes. Second, considering that this is an exploratory study, we did not correct for multiple testing for various macula and optic disc subregions, which may lead to an inevitable increase in false discovery rate. Third, given the sitting position required to perform OCTA, the OCTA examination could not be performed in the sleeping or supine position. During episodes of apnea and hypopnea events, dynamic vascular changes cannot be captured. Also, changes in body position, light stimulation during transfer, etc., may have some impact on OCTA quantification. Future studies with larger populations and more accurate and reproducible OCTA algorithms are needed to further investigate the effects of OSA on retinal and optic disc microcirculation.

In conclusion, microcirculation blood flow and overnight RNFL thickness change around the optic disc areas might emerge in OSA patients. Although there may be physiological fluctuations, aberrant diurnal changes may signal a decrease in microenvironmental stability that may be associated with the development of various ocular diseases such as glaucoma. Other vessel density (VD) variables, especially macular VDs and FAZ parameters, are relatively stable in the eyes of patients with OSA of different severity.

## Data availability statement

The raw data supporting the conclusions of this article will be made available by the authors, without undue reservation. The data supporting this study are available upon request from Prof. XS (e-mail: drxuanshi@163.com).

## Ethics statement

The studies involving human participants were reviewed and approved by the Human Research Ethics Committee of Peking University People’s Hospital. The patients/participants provided their written informed consent to participate in this study.

## Author contributions

XS, J-FQ, and FH designed this study. XS, G-SS, and YC defined the criteria for inclusion and exclusion. W-BL, MZ, LZ, G-SS, and YC performed examinations. Y-TJ and YC analyzed and interpreted the patient data, and YC, Y-TJ, and MZ drafted this manuscript and made the figures and tables. XS, FH, and M-WZ modified the manuscript. All authors read and approved the final manuscript.

## Funding

This study was supported by the National Natural Science Foundation of China (Grant No. 81970815), and National Key R&D Program of China, No. 2020YFC2008200.

## Acknowledgments

The authors are grateful to all the subjects who participated in this study.

## Conflict of interest

The authors declare that the research was conducted in the absence of any commercial or financial relationships that could be construed as a potential conflict of interest.

## Publisher’s note

All claims expressed in this article are solely those of the authors and do not necessarily represent those of their affiliated organizations, or those of the publisher, the editors and the reviewers. Any product that may be evaluated in this article, or claim that may be made by its manufacturer, is not guaranteed or endorsed by the publisher.
